# Route efficiency in spatial navigation as an early indicator of cognitive decline in amnestic mild cognitive impairment

**DOI:** 10.3389/fnagi.2026.1797674

**Published:** 2026-05-08

**Authors:** Nicole Rogers, Carol Dazil SanMartín, Jamileth More, César Romero, Daniela Paz Ponce, José Luis Valdés, María Isabel Behrens

**Affiliations:** 1 Departamento de Neurociencia, Facultad de Medicina, Universidad de Chile, Santiago, Chile; 2 Centro de Neurociencias, Clínica Universidad de los Andes, Santiago, Chile; 3 Instituto de Nutrición y Tecnología de los Alimentos (INTA), Universidad de Chile, Santiago, Chile; 4 Centro de Investigación Clínica Avanzada (CICA) Facultad de Medicina-Hospital Clínico Universidad de Chile, Santiago, Chile; 5 Departamento de Neurología y Neurocirugía, Hospital Clínico Universidad de Chile, Universidad de Chile, Santiago, Chile; 6 Laboratorio de Psiquiatría Traslacional, Departamento de Psiquiatría Norte, Universidad de Chile, Santiago, Chile; 7 Instituto de Neurociencia Biomédica, Facultad de Medicina, Universidad de Chile, Santiago, Chile; 8 Departamento de Neurología y Psiquiatría, Clínica Alemana, Universidad del Desarrollo, Santiago, Chile

**Keywords:** Alzheimer’s disease, mild cognitive impairment, Montreal cognitive assessment, spatial memory, spatial navigation, virtual Morris water maze, virtual navigation task

## Abstract

**Introduction:**

The hippocampus is one of the brain regions most affected by neurodegeneration in Alzheimer’s disease (AD). This structure and its neural circuits are critically involved in spatial learning and memory. Poor spatial navigation performance in virtual environments such as the Virtual Morris Water Navigation Task (VMWNT) may precede other clinical findings in amnestic mild cognitive impairment (aMCI), a pre-dementia stage of AD. To explore this idea, we correlated aMCI and cognitively healthy control (HC) performance in the VMWNT with Montreal Cognitive Assessment (MoCA) and Memory Index Score (MoCA-MIS), testing whether navigation-derived metrics are associated with cognitive performance within a framework of shared medial temporal lobe vulnerability.

**Methods:**

Thirty-eight participants (18 aMCI, 20 HC) were assessed for neurologic evaluation and VMWNT performance. Neuropsychological tests, including MoCA, MoCA-MIS, Clinical Dementia Rating (CDR), CDR sum of boxes (CDR-SOB) and AD8, were performed. Group differences were assessed using Mann–Whitney U tests on participant-level means and complemented with mixed-effects models to account for the repeated-measures structure of trial-level data. All behavioral parameters obtained in the VMWNT were reduced to a single variable, “Route Efficiency,” through Principal Component Analysis (PCA) on five navigation variables, with the aim to correlate spatial memory performance with clinical findings.

**Results:**

Significant differences in VMWNT performance were observed. aMCI patients displayed longer path lengths (*p* = 0.006), more quadrant crossings (*p* = 0.010), reduced time in target quadrant (*p* < 0.001), and fewer target crossings (*p* = 0.006), with group differences most evident in Stage 2; Stage 3 showed no significant differences for most variables. Route Efficiency was significantly lower in aMCI than HC (*p* < 0.001); correlations with MoCA and MoCA-MIS were observed across the full sample but were nonsignificant within groups. Stage 3 allocentric navigation did not reveal consistent group differences. Using nested cross-validation, the AUC for aMCI detection was 0.78 (95% CI: 0.63–0.91).

**Discussion:**

Group differences in Route Efficiency and correlations with cognitive measures were observed, though these associations primarily reflected between-group rather than individual variation. Allocentric significant findings were not observed. These preliminary results support further exploration of virtual navigation paradigms in clinical settings.

## 1 Introduction

Mild Cognitive Impairment (MCI) is a neurological condition characterized by cognitive deficits that exceed what is expected for an individual’s age and educational background, while their independence in daily functioning is largely preserved and without significant impairment (Petersen et al., 1999). Among its subtypes, amnestic type MCI (aMCI) has a higher rate of progression to Alzheimer’s disease (AD), with an estimated annual progression rate of 10–15% (Petersen et al., 1999; Farias et al., 2010; Petersen et al., 2014; Vos et al., 2013). This elevated risk underscores the importance of identifying early cognitive markers associated with progression to AD.

In AD, pyramidal neurons in the entorhinal cortex and hippocampus are the most susceptible to early neuronal death (Braak and Braak, 1995). Structural MRI studies have demonstrated that hippocampal and entorhinal atrophy in early AD and MCI are associated with episodic memory impairment and predict cognitive decline (Jack Jr et al., 1999; de Toledo-Morrell et al., 2000). This phenomenon is correlated with symptoms that functionally rely on medial temporal lobe integrity, such as impairment in the acquisition of new memories, difficulties regarding learning of new information, challenges in retrieving recently acquired memories, and deterioration of spatial memory (Scoville and Milner, 1957; O’Keefe and Nadel, 1978; Plácido et al., 2021).

Spatial memory can be encoded using distinct reference frames. Egocentric representations encode space relative to the individual and are supported by parietal regions, whereas allocentric representations encode object-to-object relationships independent of the observer and depend critically on medial temporal lobe structures, particularly the hippocampus. Both reference frames have been investigated using virtual navigation paradigms (Burgess et al., 2002) and have been shown to be differentially impaired in amnestic mild cognitive impairment using virtual reality paradigms (Weniger et al., 2011). A recent meta-analysis examining spatial navigation performance in Alzheimer’s disease and MCI populations reported differences across spatial strategies, suggesting that allocentric and egocentric tasks may capture partially distinct aspects of navigation impairment (Serino et al., 2025). Together, these findings suggest that different spatial navigation components may be differentially affected in the prodromal stages of Alzheimer’s disease.

The overlap between spatial navigation and episodic memory systems has been emphasized by models proposing shared hippocampal mechanisms underlying both processes (Buzsáki and Moser, 2013; Clark et al., 2005). Converging evidence from lesion studies and virtual navigation paradigms supports a central role of medial temporal structures, particularly the hippocampus, in allocentric spatial encoding (Eichenbaum et al., 1999; Burgess et al., 2002; Goodrich-Hunsaker et al., 2010).

Despite this growing body of evidence, spatial navigation abilities are not routinely assessed in clinical cognitive screening. Widely used instruments such as the Montreal Cognitive Assessment (MoCA) primarily evaluate global cognition and verbal episodic memory, without directly probing spatial navigation performance.

Studies conducted with physical, real-world labyrinths suggest that navigation and spatial memory impairment occur early in the development of AD (Hort et al., 2007). These findings are consistent with preclinical studies, which have demonstrated that early tau pathology in the entorhinal cortex is associated with deficits in spatial navigation tasks (Koike et al., 2024). Subsequent research has assessed early spatial memory impairments in MCI patients through virtual mazes (Mohammadi, 2018), demonstrating comparable sensitivity to real-world navigation tasks (Laczó et al., 2012; da Costa et al., 2022). Together, this supports the rationale for evaluating whether performance in a virtual navigation paradigm relates to established cognitive measures in aMCI.

The present study tests whether spatial navigation performance correlates with episodic memory measures (MoCA and MoCA-MIS) in cognitively healthy older adults and individuals with aMCI, based on the hypothesis that both functions share medial temporal lobe substrates and may be jointly affected in early neurodegeneration. In addition, we explored whether navigation-derived metrics could distinguish between diagnostic groups.

## 2 Materials and methods

### 2.1 Participants

Forty-six subjects were recruited, of whom eight were excluded: two due to poorly controlled type 2 diabetes mellitus and six because of an uncertain clinical cognitive diagnosis. The remaining 38 participants were included in the study and provided written informed consent approved by the Ethics Committee of Hospital Clínico Universidad de Chile (Acta AP-150, 122-2015). Cognitively healthy older adults (*n* = 20) were recruited from the community-dwelling older population of Santiago, Chile. Eligible subjects were required to be 60 years or older, with normal or corrected hearing and vision, and clinically stable chronic metabolic conditions (e.g., type 2 diabetes mellitus, hypertension). Patients (*n* = 18) were evaluated by two neurologists with extensive experience in treating patients with memory disorders (NR and MIB; Table 1).

**Table 1:** Demographic and clinical characteristics of the study participants.

Variable	Total (*n* = 38)	HC (*n* = 20)	aMCI (*n* = 18)	*p*
Age (years ± SE)	74.4 ± 1.1	73.0 ± 1.5	76.1 ± 1.7	0.17
Women, % (*n*)	63.2% (24)	75.0% (15)	50.0% (9)	0.11
Education (years ± SE)	12.4 ± 0.7	12.4 ± 0.7	12.4 ± 0.8	0.99
MoCA (score ± SE)	24.7 ± 0.7	28.0 ± 0.4	21.1 ± 0.6	<0.001
MoCA-MIS (score ± SE)	10.8 ± 0.7	14.1 ± 0.2	7.2 ± 0.8	<0.001
AD8 (score ± SE)	2.6 ± 0.4	0.6 ± 0.3	4.7 ± 0.4	<0.001
CDR (global)	0.2 ± 0.0	0.0 ± 0.0	0.5 ± 0.0	<0.001
CDR-SOB	1.03 ± 0.21	0.05 ± 0.03	2.11 ± 0.29	<0.001

HC = healthy controls; aMCI = amnestic mild cognitive impairment. Values are presented as mean ± SE. MoCA, Montreal cognitive assessment; MIS, memory index score; AD8, eight-item Informant interview to differentiate aging and dementia; CDR, clinical dementia rating; CDR-SOB, clinical dementia rating–sum of boxes.

### 2.2 Neuropsychological tests and aMCI classification

Neuropsychological tests were administered to evaluate cognitive functions, as well as the informants’ perception of these functions. The assessment included the Montreal Cognitive Assessment (MoCA), validated in Chile (Nasreddine et al., 2005; Delgado et al., 2019); the Clinical Dementia Rating (CDR; Morris, 1993) and its Sum of Boxes (CDR-SOB; O’Bryant et al., 2008); and the Eight-item Informant Interview to Differentiate Aging and Dementia (AD8), an informant-based questionnaire (Galvin et al., 2005; Muñoz et al., 2010). The Memory Index Score (MoCA-MIS) was derived from the MoCA (Julayanont et al., 2014).

Only patients with amnestic mild cognitive impairment (aMCI) were included in this study, diagnosed clinically by two neurologists (NR and MIB) according to the criteria established by Petersen et al. (1999): subjective memory complaint, objective memory impairment, preserved general cognitive function, intact activities of daily living, and absence of dementia. All aMCI participants had a global CDR of 0.5, mean CDR-SOB of 2.11 ± 0.29, and mean AD8 of 4.7 ± 0.4 (Table 1). Patients with non-amnestic MCI, active inflammatory or autoimmune diseases, active oncological conditions, or a history of kidney and/or liver disease were excluded. The control group consisted of individuals without cognitive complaints, with a global CDR of 0.

### 2.3 Virtual Morris water navigation task

**Figure 1:**
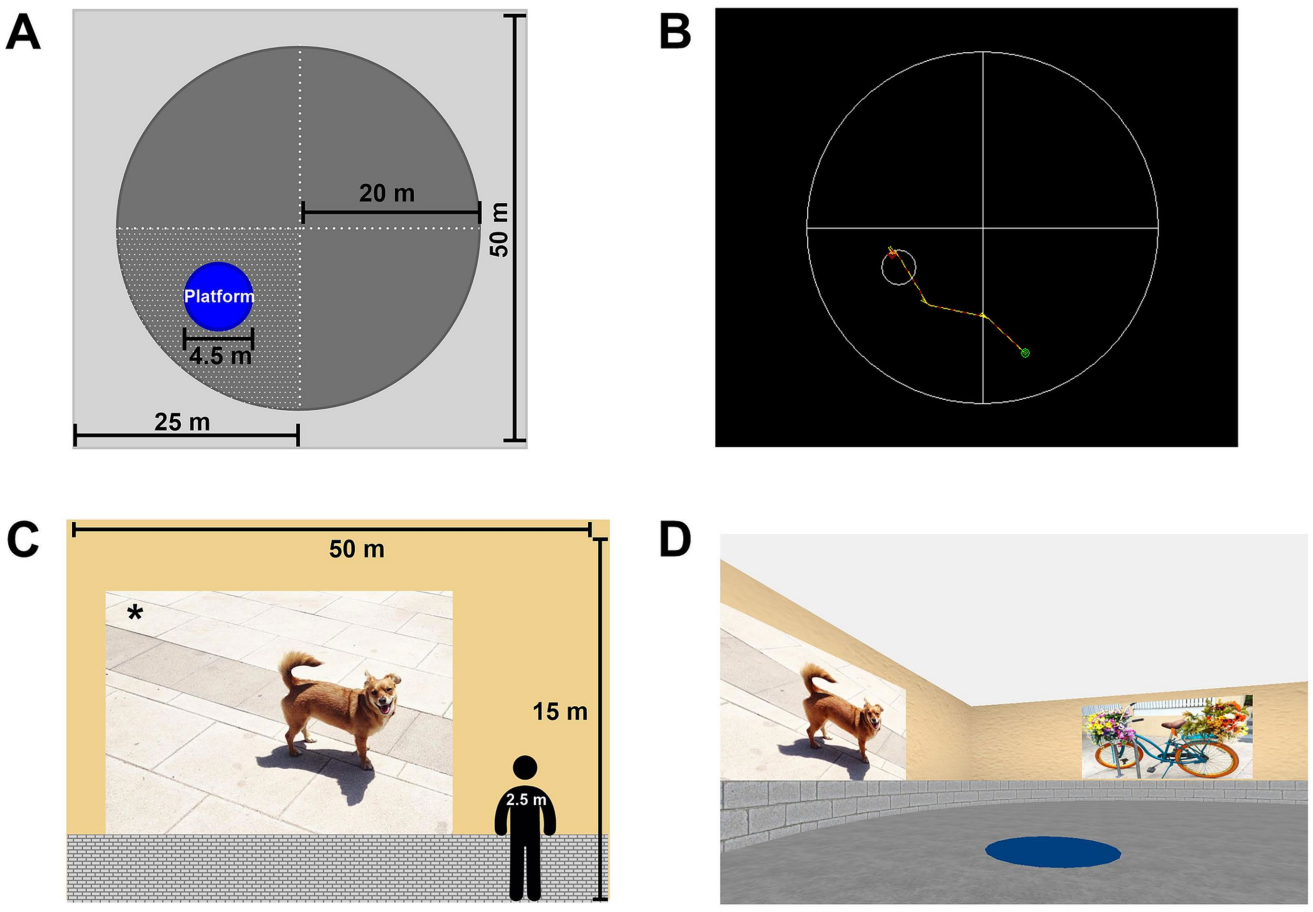
Virtual maze design and visual environment. **(A)** Top-down view of the virtual arena showing overall dimensions, circular navigation area, platform (target) location, platform size, and platform quadrant. **(B)** Representative trajectory output from the analysis software illustrating participant navigation within the arena. **(C)** Lateral view of the virtual room illustrating wall dimensions and the position of the task-relevant visual cue. **(D)** Screenshot of the virtual maze from the participant’s perspective, showing the visible platform and surrounding visual cues during task execution. The asterisk (*) denotes a task-relevant visual cue whose image content varies across task stages and trials. All measurements are expressed in meters.

To evaluate spatial memory, we used a virtual version of the Morris water maze implemented in the CG Arena software (Experiment Editor v2.05; Jacobs et al., 1997; Thomas et al., 2001). The virtual environment consisted of a computer-generated circular arena (radius = 20 metres, m) embedded within a square room (50 × 50 m) containing distal visual cues on the surrounding walls. The arena walls and floor contained no local cues distinguishing specific spatial locations. The target platform had a diameter of 4.5 m (Figure 1). All dimensions refer to virtual units.

Participants were seated in front of a 21-inch computer screen at a distance of 50 cm, in an isolated room designated for this purpose. They navigated the environment from a first-person perspective using a standard handheld video-game joystick. The task consisted of three consecutives stages of seven consecutive trials, each of them with a maximum duration of 60 s. Stage 1 was preceded by a 2-min familiarization period. Participants were instructed to locate the platform as quickly and accurately as possible.

In Stage 1, participants navigated the maze to find a visible platform. Each trial began from a different starting position, while the platform remained at a fixed, visible location. Trials ended when the participant reached the platform or when 60 s had elapsed; platform arrival was indicated by an auditory cue. An inter-trial resting period of 10 s was imposed between trials, during which no visual cues or platform were displayed.

In Stage 2, participants were required to locate a hidden platform using distal visual cues on the maze walls. All trials began from the same starting position, and the platform remained at a fixed hidden location, different from that used in Stage 1. Inter-trial periods were identical to Stage 1. Trials not completed within 60 s were terminated, latency was recorded as 60 s, and the platform became visible at trial completion without further navigation.

In Stage 3, the platform was hidden and participants began each trial from variable starting positions. Platform location and visual cues differed from those used in Stages 1 and 2. Trial termination rules were identical to Stage 2.

All task parameters, including platform coordinates, cue configuration, trial duration, and starting position constraints, were predefined and held constant across participants. The XY coordinates of each navigation trajectory were sampled at 15 Hz, allowing full reconstruction of movement paths, and were stored for subsequent analysis.

For each trial, the software computed six spatial navigation variables: (1) path length, representing the virtual distance traveled until platform arrival or trial termination; (2) latency, defined as the time elapsed until platform arrival, or 60 s in unsuccessful trials; (3) path distance ratio, calculated as the observed path length divided by the shortest possible Euclidean distance to the platform; (4) quadrant crossings, defined as the number of times participants crossed between the four equal quadrants of the circular arena; (5) time in target quadrant, the percentage of trial time spent in the platform quadrant; and (6) target crossings, indicating successful platform arrivals within 60 s.

### 2.4 Statistical analysis

Group differences in demographic and neuropsychological variables were assessed using appropriate parametric and non-parametric tests. Navigation performance was compared between groups using Mann–Whitney U tests on participant-level means, both globally (collapsed across all 21 trials) and within each stage separately. Values presented in tables and figures represent participant-level means ± standard error (SE).

In order to account for the repeated-measures structure of the data, linear mixed-effects models were used as a complementary analysis preserving the full trial-level data structure (7 trials per stage per participant). Path length, path distance ratio, and time in target quadrant were log-transformed (log[x + 1]) prior to modeling to reduce distributional skewness. Quadrant crossings were analyzed using mixed-effects Poisson regression, and target crossings using mixed-effects logistic regression. Latency was not included in the mixed-effects analysis due to censoring at 60 s; group differences in time-to-platform were evaluated using Kaplan–Meier survival curves and log-rank tests. For each outcome, models were fitted separately by stage, including group (aMCI vs. control), trial number within stage (1–7, treated as categorical), and their interaction as fixed effects, with a random intercept for each participant. Full mixed-effects model outputs, including group coefficients, 95% confidence intervals, and random-effect variances, are provided in Supplementary Table S3.

Given that multiple navigation variables showed partially overlapping group differences, a Principal Component Analysis (PCA) was conducted on standardized participant-level means of five continuous navigation variables: path length, latency, path distance ratio, quadrant crossings, and time in target quadrant. These five variables represent the full set of continuous navigation measures provided by the VMWNT software; Target crossings was excluded as a binary outcome (success/failure per trial) rather than a continuous measure. The ratio of subjects to variables (37:5 = 7.4) met minimum recommendations for stable PCA loadings. The first principal component (PC1) was retained as it accounted for the largest proportion of shared variance (53.5%; eigenvalue = 2.68), and was inverted to create a composite variable termed Route Efficiency, where higher values reflect better performance. Full PCA loadings and variance explained are provided in Supplementary Table S4.

Correlations between Route Efficiency and neuropsychological scores (MoCA and MoCA-MIS) were assessed using Spearman’s rank correlation. Exact *p*-values are reported throughout. The potential discriminative capacity of Route Efficiency metric was evaluated through ROC analysis with nested 5-fold cross-validation (10 repetitions). In each fold, PCA was computed on the training set only, and test data were projected onto the resulting PC1 loadings. A logistic regression model using PC1 as predictor was trained and applied to the held-out set. The final AUC was averaged across all folds and repetitions, with 95% CI estimated via bootstrap (1,000 iterations).

Analyses were performed using Stata 17 (StataCorp LLC) and R 4.3.2; figures were produced using GraphPad Prism. Analysis scripts are available upon request.

## 3 Results

### 3.1 Demographic and neuropsychological data

HC participants scored an average of 28.0 ± 0.4 points on the MoCA, while individuals with aMCI scored 21.1 ± 0.6 points. HC obtained a mean MoCA-MIS score of 14.1 ± 0.2, whereas aMCI patients scored 7.2 ± 0.8. HC demonstrated a mean AD8 score of 0.6 ± 0.3, while aMCI patients averaged 4.7 ± 0.4. There were no significant differences between groups in age, sex, or education, nor in the frequency of hypertension, type 2 diabetes mellitus, history of concussion, alcohol use, or benzodiazepine use (Table 1; Supplementary Table S1).

### 3.2 Performance in the VMWNT

**Figure 2:**
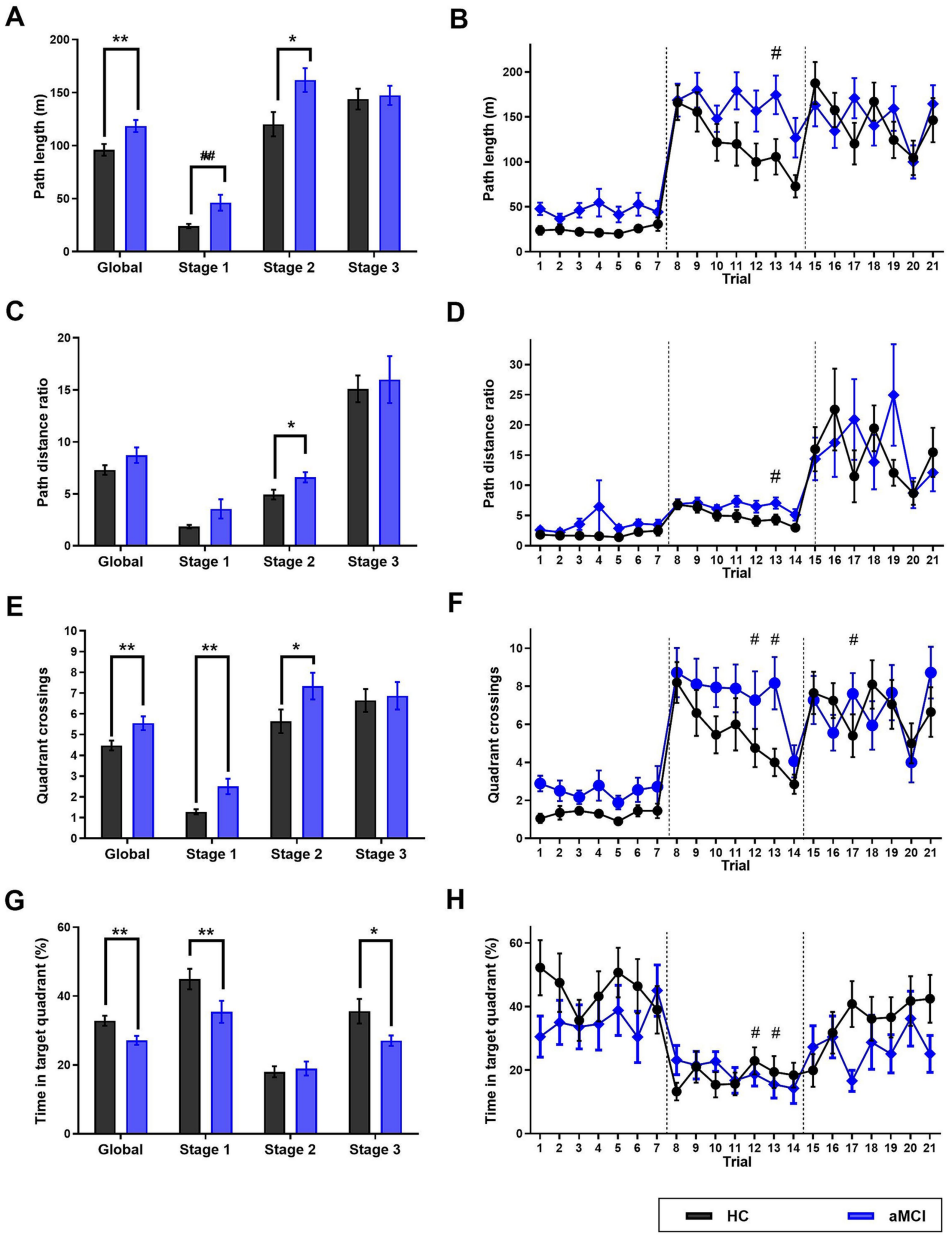
Navigation performance in the Virtual Morris Water Navigation Task (VMWNT) by group and stage. Data from individuals with amnestic mild cognitive impairment (aMCI; *n* = 18) and cognitively healthy controls (HC; *n* = 20). **(A)** Mean path length by stage. **(B)** Path length across trials. **(C)** Mean path distance ratio by stage. **(D)** Path distance ratio across trials. **(E)** Mean quadrant crossings by stage. **(F)** Quadrant crossings across trials. **(G)** Mean time in target quadrant by stage. **(H)** Time in target quadrant across trials. Data are presented as mean ± SE. Group comparisons in panels **A**, **C**, **E**, and **G** were performed using Mann–Whitney *U* tests. Significant group × trial interactions in panels **B**, **D**, **F**, and **H** were identified using linear mixed-effects models. *p < 0.05, **p < 0.01 (Mann–Whitney U test); ^#^*p* < 0.05 (group × trial interaction, mixed-effects model).

**Figure 3:**
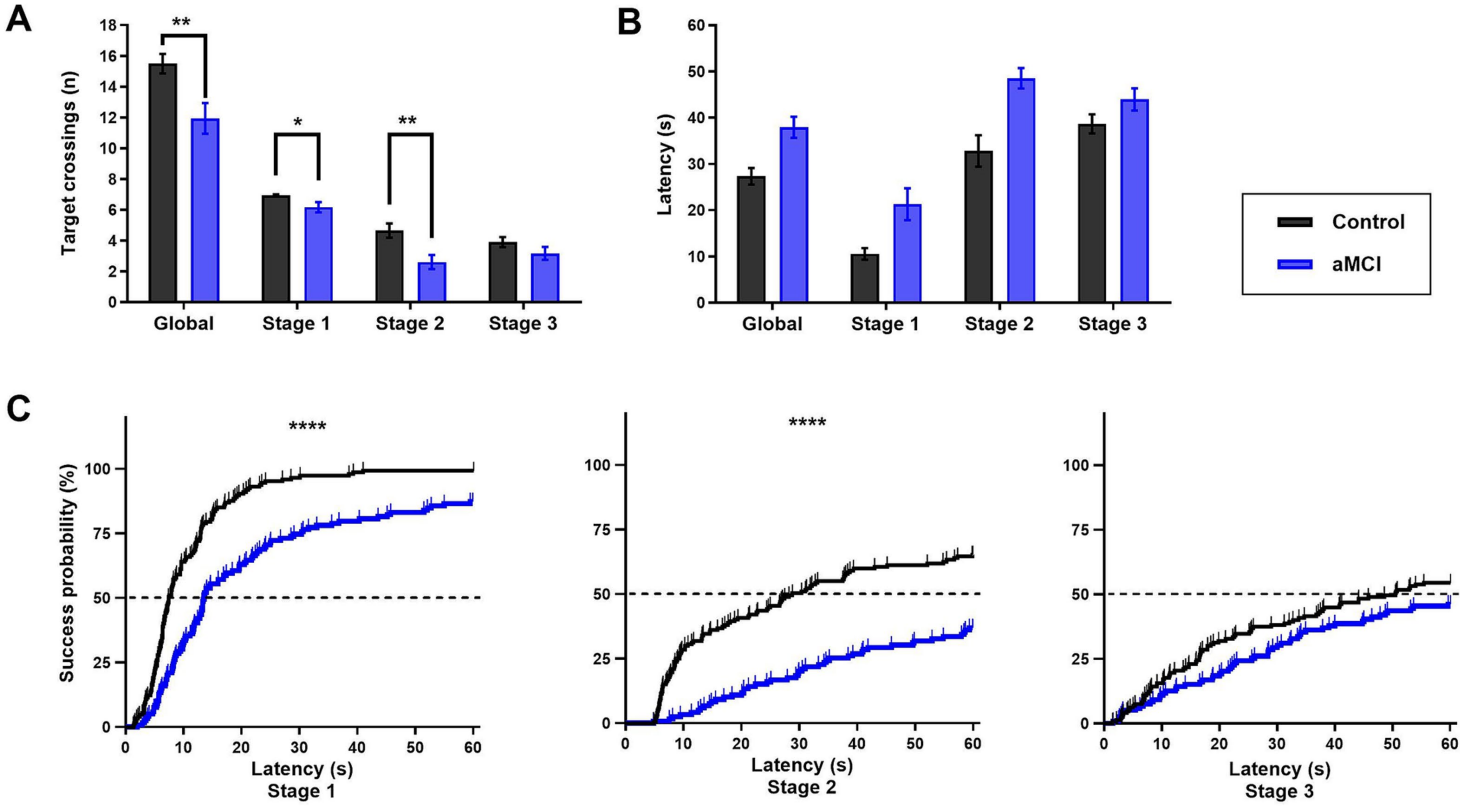
Target crossings and latency in the Virtual Morris Water Navigation Task (VMWNT). Data from individuals with amnestic mild cognitive impairment (aMCI; *n* = 18) and cognitively healthy controls (HC; *n* = 20). **(A)** Mean target crossings by stage. **(B)** Mean latency by stage. **(C)** Kaplan–Meier survival curves showing cumulative success probability by latency for each stage. The dashed line indicates 50% success probability. Group comparisons in panels A were performed using Mann–Whitney U tests. Log-rank test was used for panel C. *p < 0.05, **p < 0.01 (Mann–Whitney *U* test); ****p < 0.0001 (log-rank test).

aMCI patients showed poorer navigation performance compared to HC across multiple variables. Group differences across stages are presented in Figures 2, 3; complete statistical results are reported in Tables 2, 3. Globally, aMCI patients showed significantly longer path lengths (aMCI: 118.5 ± 5.7 m, HC: 96.0 ± 5.5 m; *p* = 0.006), more quadrant crossings (aMCI: 5.5 ± 0.3, HC: 4.5 ± 0.2; *p* = 0.010), reduced time in target quadrant (aMCI: 41.7 ± 2.3%, HC: 57.7 ± 3.1%; *p* < 0.001), and fewer target crossings (aMCI: 11.9 ± 1.0, HC: 15.5 ± 0.6; p = 0.006) compared to HC. Path distance ratio did not differ significantly between groups in the global analysis (*p* = 0.135).

**Table 2:** Navigation performance by group and stage (mean ± SE).

Variable (mean ± SE)	Global			Stage 1			Stage 2			Stage 3		
	HC	aMCI	*p*	HC	aMCI	*p*	HC	aMCI	*p*	HC	aMCI	*p*
Path length (m)	96.1 ± 5.5	118.5 ± 5.7	0.005	24.1 ± 2.1	46.2 ± 7.5	0.021	120.1 ± 11.5	161.8 ± 11.2	0.017	143.9 ± 9.9	147.4 ± 9.1	0.784
Path distance ratio^a^	7.3 ± 0.5	8.7 ± 0.7	0.141	1.8 ± 0.2	3.5 ± 0.9	0.149	4.9 ± 0.5	6.6 ± 0.5	0.026	15.1 ± 1.3	16.0 ± 2.3	0.752
Quadrant crossings	4.5 ± 0.2	5.5 ± 0.3	0.009	1.3 ± 0.1	2.5 ± 0.4	0.001	5.4 ± 0.5	7.5 ± 0.7	0.033	6.7 ± 0.5	6.7 ± 0.6	0.868
Time in target quadrant (%)	57.7 ± 3.1	41.7 ± 2.3	0.0001	82.2 ± 5.6	57.7 ± 4.3	0.001	34.8 ± 2.8	26.8 ± 3.2	0.077	56.1 ± 5.1	40.6 ± 3.0	0.021
Target crossings (*n*)	15.5 ± 0.63	11.94 ± 0.99	0.005	6.95 ± 0.05	6.17 ± 0.34	0.021	4.65 ± 0.47	2.61 ± 0.45	0.003	3.90 ± 0.32	3.17 ± 0.42	0.132

HC = healthy controls; aMCI = amnestic mild cognitive impairment. *p*-values from Mann–Whitney *U* tests.

a Path distance ratio: aMCI *n* = 17 due to one missing value; all other variables HC *n* = 20, aMCI *n* = 18. Target crossings values represent total successful platform arrivals per stage (out of 7 trials).

**Table 3:** Group × trial interactions from mixed-effects models (*β* and *p*-value).

Variable	Stage	Trial (vs. Trial 1)					
		2	3	4	5	6	7
Path length (log)	Stage 1	−0.17 (0.581)	−0.18 (0.577)	−0.27 (0.392)	−0.28 (0.369)	−0.34 (0.274)	−0.55 (0.081)
	Stage 2	0.24 (0.407)	0.39 (0.190)	0.58 (0.050)	0.56 (0.060)	0.63 (0.032)*	0.48 (0.105)
	Stage 3	−0.10 (0.838)	0.74 (0.114)	−0.19 (0.686)	0.26 (0.572)	0.04 (0.933)	0.50 (0.286)
Path ratio (log)	Stage 1	−0.07 (0.670)	0.14 (0.411)	0.07 (0.669)	0.13 (0.453)	0.05 (0.791)	0.03 (0.865)
	Stage 2	0.16 (0.499)	0.27 (0.248)	0.45 (0.060)	0.42 (0.076)	0.47 (0.047)*	0.35 (0.143)
	Stage 3	−0.22 (0.620)	0.56 (0.206)	−0.30 (0.501)	0.18 (0.681)	−0.05 (0.911)	0.02 (0.971)
Time in quadrant (log)	Stage 1	0.00 (0.987)	0.07 (0.513)	0.09 (0.415)	0.02 (0.836)	0.04 (0.736)	0.17 (0.146)
	Stage 2	−0.12 (0.095)	−0.07 (0.296)	−0.12 (0.085)	−0.16 (0.023)*	−0.16 (0.024)*	−0.12 (0.073)
	Stage 3	0.02 (0.863)	−0.26 (0.051)	−0.09 (0.506)	−0.05 (0.690)	−0.04 (0.773)	−0.20 (0.121)
Quadrant crossings	Stage 1	−0.40 (0.265)	−0.61 (0.087)	−0.25 (0.475)	−0.27 (0.487)	−0.45 (0.204)	−0.38 (0.273)
	Stage 2	0.14 (0.378)	0.32 (0.063)	0.21 (0.204)	0.36 (0.037)*	0.65 (<0.001)*	0.29 (0.164)
	Stage 3	−0.22 (0.220)	0.39 (0.025)*	−0.26 (0.132)	0.13 (0.428)	−0.17 (0.374)	0.32 (0.055)
Target crossings	Stage 1	–	–	–	–	–	–
	Stage 2	−0.84 (0.478)	−0.83 (0.489)	−0.80 (0.498)	−1.92 (0.125)	−1.51 (0.217)	−1.77 (0.169)
	Stage 3	0.52 (0.590)	−0.39 (0.688)	0.03 (0.973)	−0.89 (0.367)	−0.39 (0.691)	−0.26 (0.789)

*β* = regression coefficient for the aMCI × trial interaction (reference: Trial 1, control group). Values in parentheses are *p*-values. *p < 0.05. Target crossings Stage 1: not estimable due to perfect prediction (platform visible, near-universal success). Path distance ratio: aMCI *n* = 17. Convergence issues noted for path length and path ratio Stage 3.

When analyzed by stage, significant group differences were observed primarily in Stage 2, with aMCI patients showing longer path lengths (*p* = 0.018), higher path distance ratio (*p* = 0.026), more quadrant crossings (*p* = 0.033), and fewer target crossings (*p* = 0.004) compared to HC. In Stage 3, most variables did not differ significantly between groups, with the exception of time in target quadrant (*p* = 0.021; Table 2). Stage 1 showed some between-group differences in univariate analyses, although group × trial interactions were not significant in mixed-effects models.

To account for the repeated-measures structure of the data, linear mixed-effects models were fitted for each outcome. For path length, the main effect of group was significant in Stage 1 (*β* = 0.771, *p* = 0.002), but not in Stages 2 or 3. Importantly, group × trial interactions were not significant for path length in any stage, suggesting that both groups showed similar learning rates across trials despite baseline differences. For quadrant crossings (Poisson mixed model), significant group × trial interactions were observed at specific trials in Stage 2 (trials 11 and 12, *p* < 0.05), indicating differential learning patterns between groups. Similar interactions were found for time in the target quadrant in Stage 2. For path distance ratio, a significant group × trial interaction was observed in Stage 3, although this should be interpreted with caution given the absence of main group effects in this stage. Target crossings were analyzed using mixed-effects logistic regression. The main effect of group was significant in the global model (*p* < 0.001), but group × trial interactions were not significant (Table 3).

### 3.3 Latency: survival analysis

Cumulative success probability was compared using Kaplan–Meier survival curves and log-rank tests (Figure 3C). aMCI patients showed significantly lower probability of reaching the platform in Stage 1 (log-rank *p* < 0.0001) and Stage 2 (log-rank *p* < 0.0001). In Stage 2, fewer than half of aMCI patients successfully located the platform, as indicated by a median latency that was not reached in this group. No significant differences were observed in Stage 3 (log-rank *p* = 0.131).

### 3.4 Route efficiency and correlation with cognitive status

**Figure 4:**
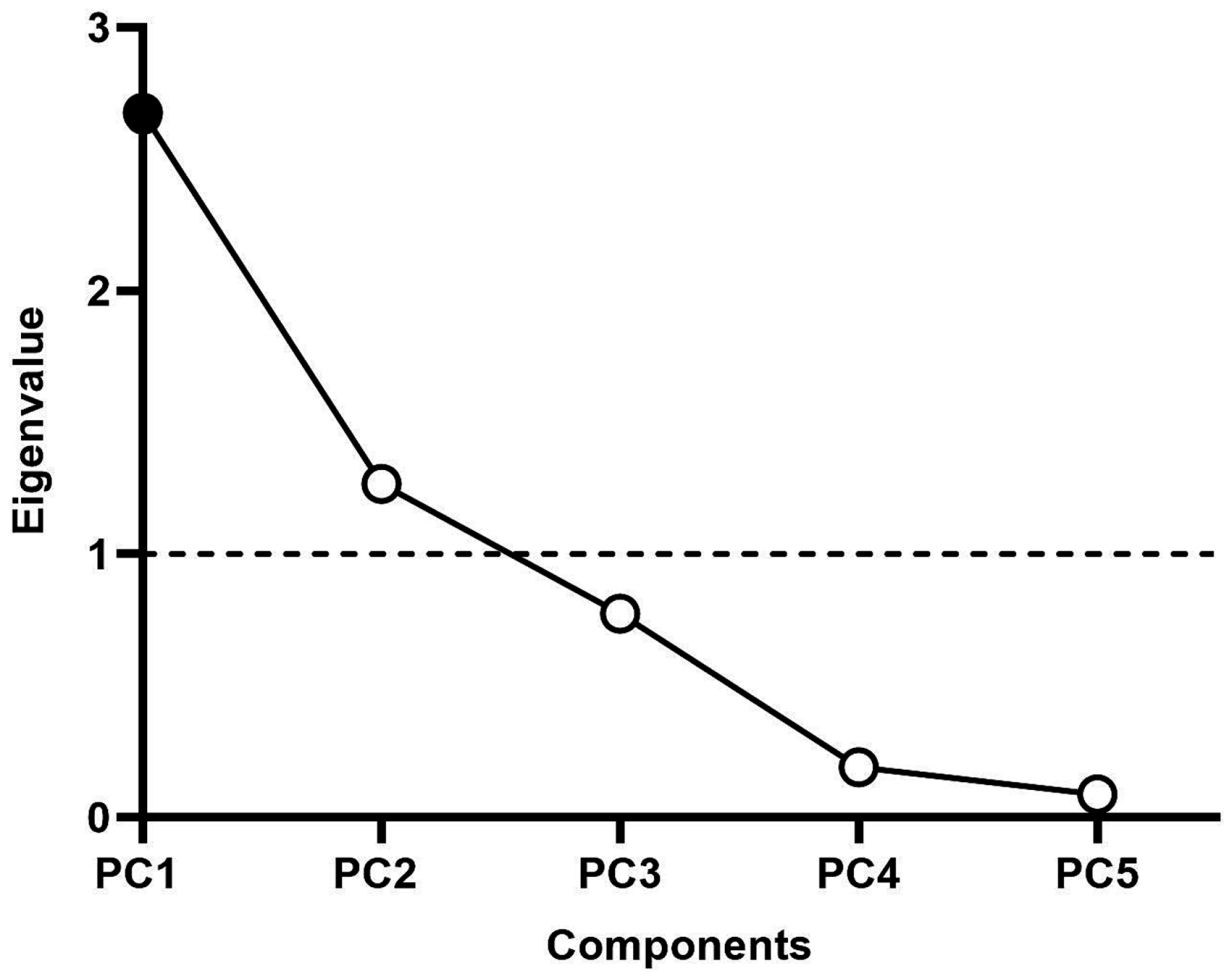
Scree plot of principal component analysis. The dashed line indicates the Kaiser criterion (eigenvalue = 1). PC1 was retained for further analysis (eigenvalue = 2.68, proportion of variance explained = 53.5%). *n* = 37.

Given that multiple navigation variables showed overlapping group differences, a Principal Component Analysis (PCA) was conducted on standardized participant-level means of five continuous variables: path length, latency, path distance ratio, quadrant crossings, and time in target quadrant (*n* = 37). PC1 accounted for 53.5% of the variance (eigenvalue = 2.68) and was the only component exceeding the Kaiser criterion (Figure 4, Supplementary Table S4). PC1 was inverted to create Route Efficiency, where higher values reflect better performance.

**Figure 5:**
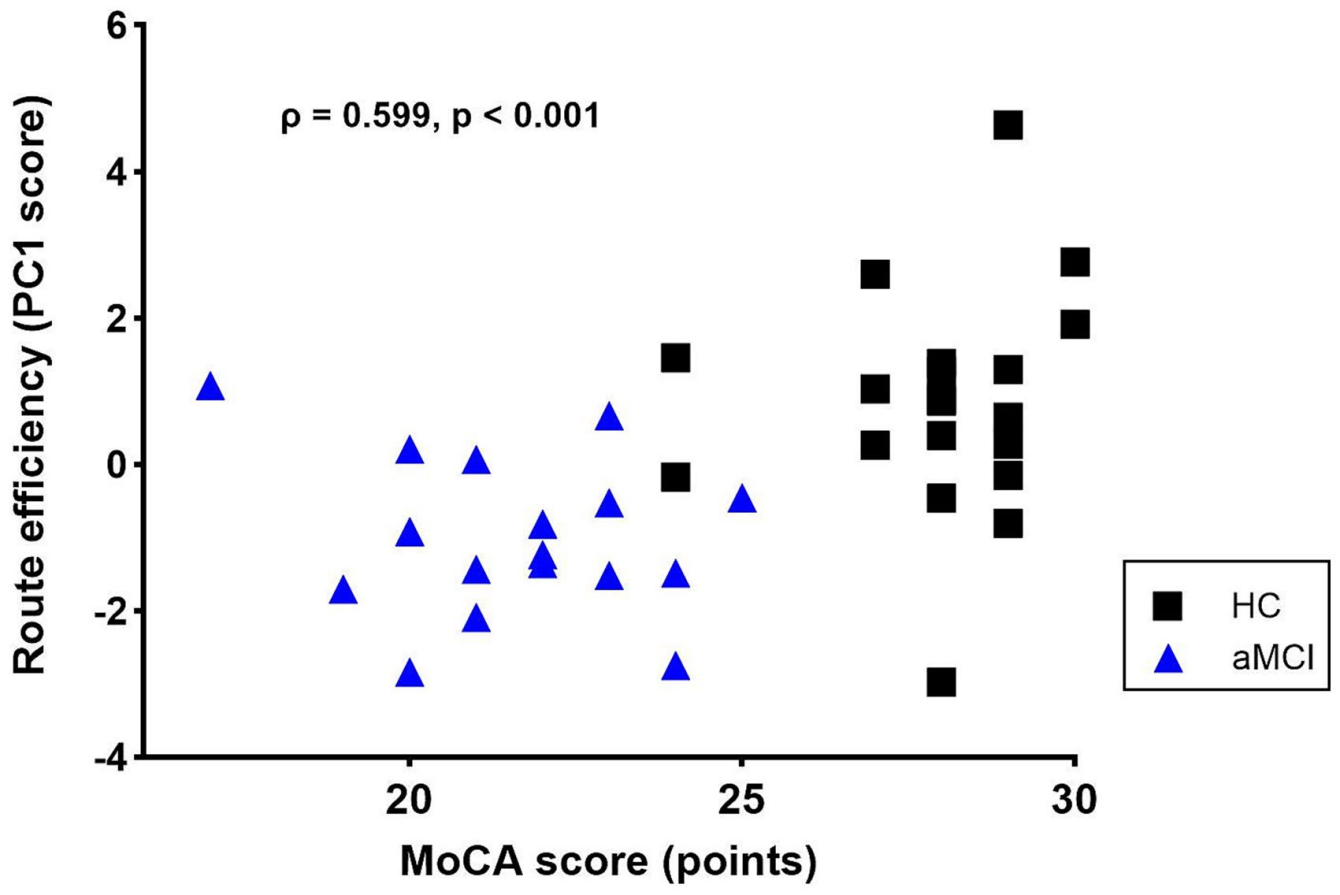
Association between route efficiency and MoCA score (*n* = 37). Spearman correlation: *ρ* = 0.599, *p* < 0.001. HC = healthy controls; aMCI = amnestic mild cognitive impairment.

Route Efficiency was significantly higher in HC compared to aMCI (HC: 0.86 ± 0.35, aMCI: −1.01 ± 0.27; *z* = 3.749, *p* < 0.001; d = 1.39, post-hoc power > 0.99). This difference remained significant after adjusting for sex, age, and education (*β* = −1.48, *p* = 0.006). Route Efficiency correlated significantly with MoCA (*ρ* = 0.599, p < 0.001) and MoCA-MIS (*ρ* = 0.553, *p* < 0.001) (Figure 5). Within-group correlations were not significant in either HC (MoCA: *ρ* = 0.162, *p* = 0.496; MoCA-MIS: *ρ* = 0.072, *p* = 0.764) or aMCI (MoCA: *ρ* = 0.325, *p* = 0.204; MoCA-MIS: *ρ* = 0.234, *p* = 0.366). Partial correlations controlling for group were also not significant (MoCA: partial *r* = 0.230, *p* = 0.176; MoCA-MIS: partial *r* = 0.110, *p* = 0.522), indicating that global correlations reflect between-group differences (Supplementary Table S5).

**Figure 6:**
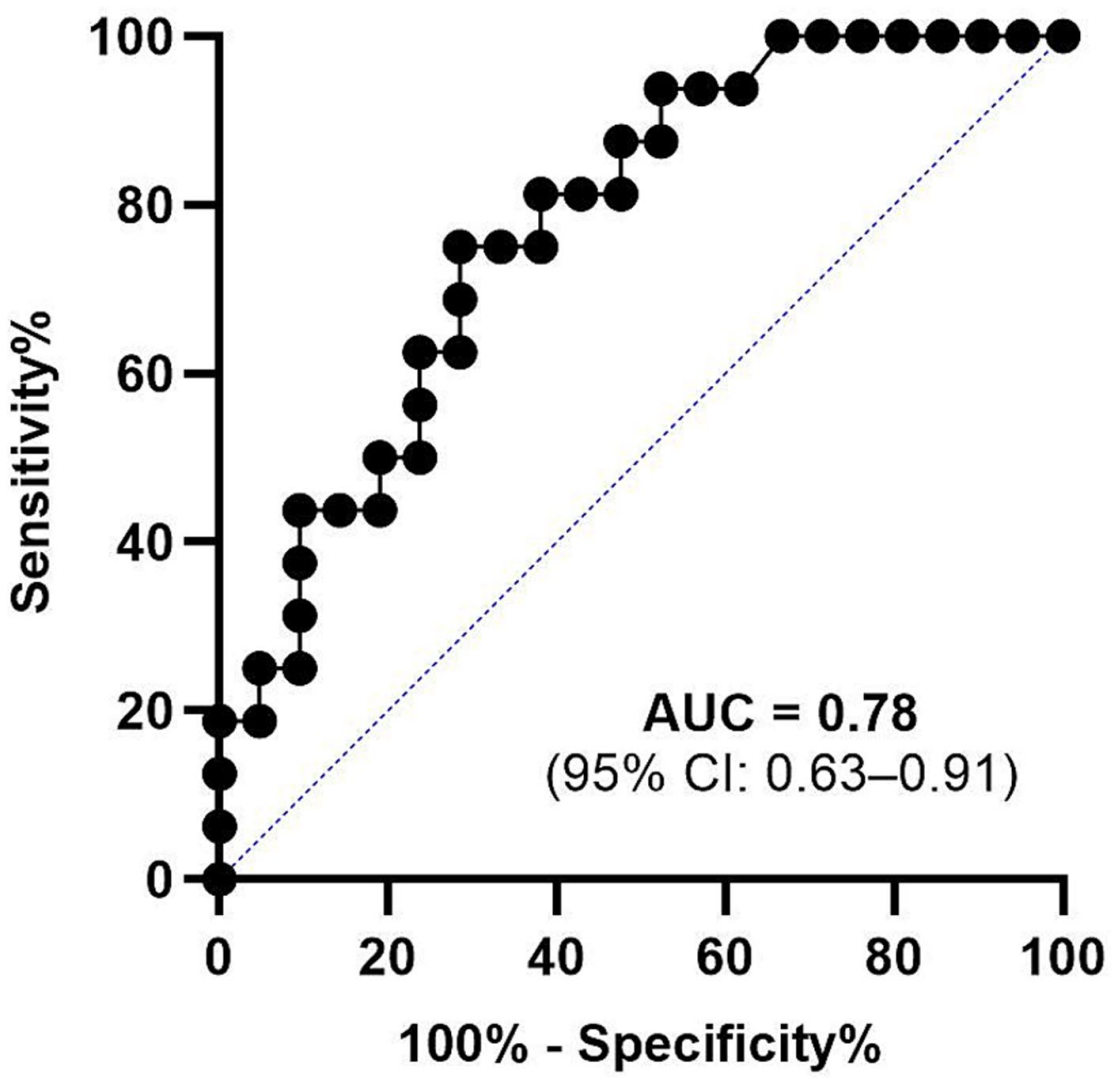
ROC curve for route efficiency using nested cross-validation (*n* = 37). AUC = 0.78 (95% CI: 0.63–0.91). The diagonal line indicates chance performance. HC = healthy controls; aMCI = amnestic mild cognitive impairment.

ROC analysis with nested cross-validation yielded an AUC of 0.78 (95% CI: 0.63–0.91; Figure 6). ROC analyses on individual variables yielded comparable AUCs (range: 0.64–0.84; Supplementary Table S6), confirming that the composite does not artificially inflate classification performance.

## 4 Discussion

We designed a behavioral test that engages memory processes mediated by medial temporal lobe structures and observed that aMCI patients exhibited poorer performance in the VMWNT compared to HC. This reduced performance correlated significantly with neuropsychological scales assessing cognitive impairment severity across all participants. These findings align with previous studies investigating behavioral deficits in MCI patients (Laczó et al., 2009; Laczó et al., 2012) and are consistent with prior evidence linking spatial navigation performance to CSF biomarkers, p-tau deposition, elevated NfL levels, and medial temporal lobe atrophy (Tangen et al., 2022; Laczó et al., 2025).

The original Morris water maze, introduced in 1981, was developed to study learning processes and the acquisition of spatial memory in rodents. The task involves a pool in which rats search for a hidden platform that provides escape from the water (Morris, 1981). In humans, virtual adaptations of the Morris water maze have proven effective for assessing hippocampal function. These paradigms present fewer cultural and educational biases compared to traditional neuropsychological assessments, making them particularly suitable for diverse populations (Laczó et al., 2017; Coughlan et al., 2018).

Although impaired performance in hippocampus-dependent virtual mazes has been reported in patients with aMCI and AD, the present implementation revealed findings that merit careful consideration. First, aMCI patients performed significantly worse during Stage 1, a phase with a visible platform originally designed to identify subjects with motor or visual impairments. Efficient performance at this stage relies more on executive functions than on hippocampal integrity, as no spatial memory is required to locate a visible target. Impaired visible-platform performance has been reported primarily in conditions characterized by frontal dysfunction: depression (Cornwell et al., 2010), traumatic brain injury (Skelton et al., 2006), and alcohol dependence (Ceccanti et al., 2015), conditions that were systematically excluded in our sample. Correlations between Morris maze performance and executive function measures have been described, though mainly in younger cohorts (Korthauer et al., 2016). The role of executive functions in virtual navigation performance among patients with aMCI has not been extensively characterized, and our findings suggest this could be a productive avenue for future research.

A second finding that warrants discussion is the absence of significant group differences in Stage 3 for most navigation variables. This stage was designed to isolate allocentric navigation processes, which are reported to be preferentially affected in aMCI and AD (Parizkova et al., 2018). Given the hypothesized dependence of allocentric processing on hippocampal integrity, we expected this stage to show the largest group differences. However, Stage 3 introduced both variable starting positions and a hidden platform, along with novel visual cues which substantially increased task demands relative to earlier stages. Under these conditions, neither group achieved efficient navigation, which may have attenuated between-group differences that would otherwise emerge with extended training or reduced task complexity. The only variable distinguishing groups was time in target quadrant, suggesting that HC maintained better spatial orientation relative to the platform location despite overall poor performance. This interpretation is consistent with findings from Moffat et al. (2002), who demonstrated that older adults generally require more time and travel greater distances in virtual navigation tasks compared to younger individuals. Whether allocentric deficits in aMCI would become apparent under modified task parameters, such as extended trial duration, additional training trials, or incremental introduction of novel cues, remains to be determined in future studies. More broadly, spatial navigation typically recruits both egocentric and allocentric reference frames to varying degrees, and the boundaries between stages may not reflect strict dissociations in underlying processes. These null findings limit conclusions about allocentric-specific impairment; such interpretations should be considered preliminary.

To reduce the dimensionality of navigation performance, Route Efficiency was extracted through PCA on five continuous variables provided by the VMWNT. Route Efficiency correlated significantly with MoCA and MoCA-MIS across the full sample, consistent with previous reports linking navigation performance to cognitive measures (Parizkova et al., 2018; Nedelska et al., 2012). However, these correlations primarily reflect between-group differences rather than individual variability (Supplementary Table S5), which limits interpretation regarding shared underlying mechanisms. Within-group correlations were not significant, as expected given the limited sample sizes and restricted range within each diagnostic category. Using nested cross-validation, the AUC of 0.78 (95% CI: 0.63–0.91) suggests moderate exploratory discriminative capacity for distinguishing aMCI from HC.

There are practical advantages to detecting cognitive deficits through virtual behavioral tests. The VMWNT requires simple execution without the presence of a trained examiner, unlike formal neuropsychological testing. It may reduce biases associated with low educational attainment and test-related anxiety that can affect performance on traditional assessments. Standardization of a test like the VMWNT as a diagnostic tool would require more detailed exploration of optimal parameters, including time limits and number of trials per stage. The test is relatively time-efficient, requiring approximately 15–20 min, and demands no specialized equipment beyond a standard screen and joystick.

The study has limitations that should be acknowledged. The sample size, though adequate for detecting the primary group difference, may limit the generalizability of findings. Given the small sample (*n* = 38), PCA loadings may be unstable despite a ratio of 7.4 subjects per variable. Although nested cross-validation mitigates circularity by computing PCA within training folds, Route Efficiency may still partially embed group differences present in the data; these results should therefore be considered exploratory and require validation in a larger independent sample. No *a priori* power analysis was performed, and the study may be underpowered for complex multivariate analyses. The wide confidence interval of the cross-validated AUC (0.63–0.91) reflects these constraints. Given the modest AUC and Stage 3 yielded null findings, discriminative estimates should be considered exploratory, and the paradigm should not be interpreted as clinically diagnostic. The cross-sectional design precludes inferences about longitudinal change or predictive validity for progression to dementia. Sex distribution differed numerically between groups; however, Route Efficiency did not differ by sex after accounting for diagnosis. Future investigations with larger samples and longitudinal follow-up are needed to confirm these preliminary findings.

In summary, this study provides preliminary evidence that spatial navigation and episodic memory measures are correlated across older adults with and without aMCI, and demonstrates the feasibility of virtual navigation paradigms in clinical research settings. Clarifying the mechanisms underlying this association will require larger samples, longitudinal follow-up to assess predictive validity, neuroimaging to evaluate structural correlates, biomarker data to link navigation deficits with neurodegeneration markers, and comparison across MCI subtypes including non-amnestic presentations.

## Data Availability

The raw data supporting the conclusions of this article will be made available by the authors, without undue reservation.
