# Supplementary material for: Route efficiency in spatial navigation as an early indicator of cognitive decline in amnestic mild cognitive impairment
Source: Front Aging Neurosci. 2026 May 8;18:1797674. doi: 10.3389/fnagi.2026.1797674 (PMC13194101; doi:10.3389/fnagi.2026.1797674)
Supplement: Supplementary file 1 [file Table_1.docx]

**Supplementary Table S1. Comorbidities and medications**

|  | **Total (n = 38)** | **HC (n = 20)** | **aMCI (n = 18)** | **p** |
| --- | --- | --- | --- | --- |
| Hypertension, % (n) | 60.5 (23) | 65.0 (13) | 55.6 (10) | 0.55 |
| Diabetes mellitus type 2, % (n) | 13.2 (5) | 20.0 (4) | 5.6 (1) | 0.19 |
| HbA1c, mean % | 5.7 | 5.6 | 5.9 | 0.10 |
| Mood symptoms, % (n) | 26.3 (10) | 15.0 (3) | 38.9 (7) | 0.10 |
| SSRI use, % (n) | 26.3 (10) | 15.0 (3) | 38.9 (7) | 0.10 |
| BZP use, % (n) | 24.2 (8) | 27.8 (5) | 20.0 (3) | 0.60 |
| Dementia medication, % (n) | 15.8 (6) | 0.0 (0) | 33.3 (6) | 0.005 |
| Concussion, % (n) | 21.1 (8) | 20.0 (4) | 22.2 (4) | 0.87 |
| Alcohol use, % (n) | 44.7 (17) | 50.0 (10) | 38.9 (7) | 0.49 |

*HC = healthy controls; aMCI = amnestic mild cognitive impairment; HbA1c = glycated hemoglobin; SSRI = selective serotonin reuptake inhibitors; BZP = benzodiazepines. Group comparisons performed using chi-square tests for categorical variables and Mann-Whitney U test for HbA1c.*

**Supplementary Table S2. Distribution of CDR-SOB scores by cognitive group**

| **CDR-SOB** | **Total (n = 38)** | **HC (n = 20)** | **aMCI (n = 18)** | **p** |
| --- | --- | --- | --- | --- |
| Score, mean ± SE | 1.03 ± 0.21 | 0.05 ± 0.03 | 2.11 ± 0.29 | <0.001 |
| 0 | 18 | 18 | 0 |  |
| 0.5 | 2 | 2* | 0 |  |
| 1 | 2 | 0 | 2 |  |
| 1.5 | 5 | 0 | 5 |  |
| 2 | 2 | 0 | 2 |  |
| 2.5 | 1 | 0 | 1 |  |
| 3 | 3 | 0 | 3 |  |
| 3.5 | 2 | 0 | 2 |  |
| 5 | 1 | 0 | 1 |  |

*HC = healthy controls; aMCI = amnestic mild cognitive impairment; CDR-SOB = Clinical Dementia Rating Sum of Boxes. * Patient had 0.5 in a non-amnestic domain. Group comparison performed using Mann-Whitney U test.*

**Supplementary Table S3. Mixed-effects model results: Group effect coefficients by navigation variable and stage**

| **Variable** | **Stage** | **Group (β)** | **SE** | **95% CI** | **p** | **σ²ᵤ** | **σ²ₑ** |
| --- | --- | --- | --- | --- | --- | --- | --- |
| Path length (log) | 1 | 0.771 | 0.252 | 0.28, 1.27 | 0.002 | 0.133 | 0.470 |
|  | 2 | 0.063 | 0.240 | -0.41, 0.53 | 0.793 | 0.133 | 0.412 |
|  | 3 | -0.044 | 0.331 | -0.69, 0.61 | 0.895 | <0.001 | 1.038 |
| Path distance ratio (log) | 1 | 0.211 | 0.158 | -0.10, 0.52 | 0.183 | 0.089 | 0.142 |
|  | 2 | 0.037 | 0.191 | -0.34, 0.41 | 0.846 | 0.079 | 0.257 |
|  | 3 | -0.021 | 0.311 | -0.63, 0.59 | 0.947 | <0.001 | 0.887 |
| Time in target quadrant (log) | 1 | -0.192 | 0.083 | -0.35, -0.03 | 0.020 | 0.003 | 0.062 |
|  | 2 | 0.040 | 0.055 | -0.07, 0.15 | 0.458 | 0.005 | 0.023 |
|  | 3 | -0.013 | 0.093 | -0.19, 0.17 | 0.884 | <0.001 | 0.081 |
| Quadrant crossings* | 1 | 0.955 | 0.294 | 0.38, 1.53 | 0.001 | 0.175 | - |
|  | 2 | 0.087 | 0.177 | -0.26, 0.43 | 0.625 | 0.175 | - |
|  | 3 | -0.066 | 0.167 | -0.39, 0.26 | 0.691 | 0.127 | - |
| Target crossings** | 1 | -2.193 | 2.106 | -6.32, 1.93 | 0.298 | 7.759 | - |
|  | 2 | -0.795 | 1.001 | -2.76, 1.17 | 0.427 | 2.425 | - |
|  | 3 | -0.277 | 0.719 | -1.69, 1.13 | 0.701 | 0.397 | - |

*β = unstandardized coefficient for group (aMCI vs HC); SE = standard error; CI = confidence interval; σ²ᵤ = random intercept variance (participant); σ²ₑ = residual variance. Path length, path distance ratio, and time in target quadrant were log-transformed. *Poisson mixed model (σ²ₑ not applicable). **Logistic mixed model (σ²ₑ not applicable). Models included group × trial interaction terms (see Table 3 for interaction coefficients). Models for path length and path distance ratio in Stage 3 did not achieve full convergence; results for these models should be interpreted with caution.*

**Supplementary Table S4. Principal Component Analysis: full loading matrix and variance explained**

**A. Eigenvalues and variance explained**

| **Component** | **Eigenvalue** | **Proportion of variance** | **Cumulative** |
| --- | --- | --- | --- |
| 1 | 2.677 | 0.535 | 0.535 |
| 2 | 1.267 | 0.253 | 0.789 |
| 3 | 0.775 | 0.155 | 0.944 |
| 4 | 0.192 | 0.038 | 0.982 |
| 5 | 0.088 | 0.018 | 1.000 |

**B. Component loadings (eigenvectors)**

| **Variable** | **PC1*** | **PC2** | **PC3** | **PC4** | **PC5** |
| --- | --- | --- | --- | --- | --- |
| Path length | 0.534 | 0.319 | -0.246 | 0.334 | -0.664 |
| Latency | 0.438 | -0.555 | 0.147 | 0.605 | 0.335 |
| Path distance ratio | 0.312 | 0.325 | 0.881 | -0.146 | 0.007 |
| Quadrant crossings | 0.473 | 0.447 | -0.369 | -0.194 | 0.635 |
| Time in target quadrant | -0.450 | 0.534 | 0.073 | 0.681 | 0.210 |

*PCA was conducted on standardized (z-scored) participant-level means of five continuous navigation variables (n = 37; one missing value in path distance ratio). * PC1 was retained and inverted to create Route Efficiency (higher values = better performance). Bold values indicate PC1 loadings used to derive Route Efficiency****.***

**Supplementary Table S5. Within-group and partial correlations between Route Efficiency and cognitive measures**

| **Analysis** | **n** | **ρ / partial r** | **p** |
| --- | --- | --- | --- |
| **Within-group: HC** |  |  |  |
| Route Efficiency vs MoCA | 20 | 0.162 | 0.496 |
| Route Efficiency vs MoCA-MIS | 20 | 0.072 | 0.764 |
| **Within-group: aMCI** |  |  |  |
| Route Efficiency vs MoCA | 17 | 0.325 | 0.204 |
| Route Efficiency vs MoCA-MIS | 17 | 0.234 | 0.366 |
| **Partial correlations (controlling for group)** |  |  |  |
| Route Efficiency vs MoCA | 37 | 0.230 | 0.176 |
| Route Efficiency vs MoCA-MIS | 37 | 0.110 | 0.522 |

*ρ = Spearman correlation coefficient; partial r = Pearson partial correlation controlling for diagnostic group. HC = healthy controls; aMCI = amnestic mild cognitive impairment; MoCA = Montreal Cognitive Assessment; MoCA-MIS = MoCA Memory Index Score.*

**Supplementary Table S6. ROC analysis for individual navigation variables and Route Efficiency**

| **Variable** | **n** | **AUC** | **SE** | **95% CI** |
| --- | --- | --- | --- | --- |
| Path length | 38 | 0.76 | 0.082 | 0.60 – 0.92 |
| Latency | 38 | 0.80 | 0.072 | 0.66 – 0.94 |
| Path distance ratio | 37 | 0.64 | 0.094 | 0.46 – 0.83 |
| Quadrant crossings | 38 | 0.75 | 0.084 | 0.58 – 0.91 |
| Time in target quadrant | 38 | 0.84 | 0.064 | 0.72 – 0.97 |
| Route Efficiency | 38 | 0.78 | 0.076 | 0.63 – 0.91 |

*AUC = area under the ROC curve; SE = standard error; CI = confidence interval. ROC analyses were performed on participant-level means. Time in target quadrant AUC is reported as 1 minus the original value (0.16) to reflect higher values indicating better performance in HC. Path distance ratio n = 37 due to one missing value.*
